# Human Cytomegalovirus Tegument Protein pp65 Is Detected in All Intra- and Extra-Axial Brain Tumours Independent of the Tumour Type or Grade

**DOI:** 10.1371/journal.pone.0108861

**Published:** 2014-09-30

**Authors:** Sylwia Libard, Svetlana N. Popova, Rose-Marie Amini, Vesa Kärjä, Timo Pietiläinen, Kirsi M. Hämäläinen, Christer Sundström, Göran Hesselager, Michael Bergqvist, Simon Ekman, Maria Zetterling, Anja Smits, Pelle Nilsson, Susan Pfeifer, Teresita Diaz de Ståhl, Gunilla Enblad, Fredrik Ponten, Irina Alafuzoff

**Affiliations:** 1 Department of Pathology, Uppsala University Hospital, Uppsala, Sweden; 2 Department of Immunology, Genetics and Pathology, Uppsala University, Uppsala, Sweden; 3 Department of Clinical Pathology, Kuopio University Hospital, Kuopio, Finland; 4 Department of Neurosurgery, Uppsala University Hospital, Uppsala, Sweden; 5 Department of Oncology, Centre for Research & Development, County Council of Gävleborg, Gävle Hospital, Gävle, Sweden; 6 Department of radiation sciences, Umeå University, Umeå, Sweden; 7 Department of Radiology, Oncology and Radiation Science, Section of Oncology, Uppsala University, Uppsala, Sweden; 8 Department of Neuroscience Neurology, Uppsala University, Uppsala, Sweden; 9 Department of Women's and Children's Health, Uppsala University, Uppsala University Hospital, Uppsala, Sweden; 10 Department of Oncology-Pathology, Karolinska Institutet, Cancer Center Karolinska, Karolinska University Hospital, Stockholm, Sweden; Istituto dei tumori Fondazione Pascale, Italy

## Abstract

Human cytomegalovirus (HCMV) has been indicated being a significant oncomodulator. Recent reports have suggested that an antiviral treatment alters the outcome of a glioblastoma. We analysed the performance of commercial HCMV-antibodies applying the immunohistochemical (IHC) methods on brain sample obtained from a subject with a verified HCMV infection, on samples obtained from 14 control subjects, and on a tissue microarray block containing cores of various brain tumours. Based on these trials, we selected the best performing antibody and analysed a cohort of 417 extra- and intra-axial brain tumours such as gliomas, medulloblastomas, primary diffuse large B-cell lymphomas, and meningiomas. HCMV protein pp65 immunoreactivity was observed in all types of tumours analysed, and the IHC expression did not depend on the patient's age, gender, tumour type, or grade. The labelling pattern observed in the tumours differed from the labelling pattern observed in the tissue with an active HCMV infection. The HCMV protein was expressed in up to 90% of all the tumours investigated. Our results are in accordance with previous reports regarding the HCMV protein expression in glioblastomas and medulloblastomas. In addition, the HCMV protein expression was seen in primary brain lymphomas, low-grade gliomas, and in meningiomas. Our results indicate that the HCMV protein pp65 expression is common in intra- and extra-axial brain tumours. Thus, the assessment of the HCMV expression in tumours of various origins and pathologically altered tissue in conditions such as inflammation, infection, and even degeneration should certainly be facilitated.

## Introduction

Human cytomegalovirus (HCMV) has been associated with tumours such as primary intracerebral tumours, neuroblastoma, colorectal cancer, prostate cancer, and non-melanoma skin carcinomas in humans [1]–[6]. Particular interest has been shown for the association between the HCMV protein expression and primary, highly malignant, non-curable brain tumours such as glioblastoma (GBM) [1], [7], [8]. To our knowledge, only a few other types of brain tumours, intra- or extra-axial have been investigated regarding the HCMV protein expression [1], [8]–[12]. Interestingly, no signs of an active infection such as intranuclear inclusions have been observed in these tumours. Meanwhile, the HCMV DNA and RNA have been detected in a subset of samples that have been assessed [1], [7], [13]. Noteworthy, when the HCMV DNA was investigated in a set of GBMs, only 1 out of 80 tumour cells was shown to carry the viral DNA [14].

Recently, it has been suggested that by treating for the HCMV infection, the progression of the primary disease, GBM, is halted even though it is not significant [15]. The high prevalence of the HCMV protein expression, as reported previously in GBM, makes the HCMV an interesting therapeutic target even if only a progression related effect is achieved. Thus, currently there are ongoing studies involving the antiviral therapies as a complementary treatment of subjects with GBM [13], [15], [16].

HCMV is a member of the *Betaherpesvirinae* subfamily of the *Herpesviridae*. An HCMV virion contains about 60 virus-encoded proteins and more than 70 host-proteins. Similar to all herpes viruses, the HCMV virion has three basic structural units: capsid, tegument, and envelope. Tegument is the link between the capsid and the envelope. It contains a number of proteins, two of which (pp65 and pp71) are key regulatory proteins that are delivered into the host cell during an infection. pp65 is the most abundant virion protein and has an immunomodulatory role. It is also a major component of the dense bodies – non-infectious viral particles that are assembled during active infection [17], [18]. In contrast, pp71 is a transcriptional transactivator. HCMV can be present in the monocytes during the latent phase of an infection when virions are not produced. During this latent phase, the viral DNA is replicated in close contact with the host DNA using host-cell replication machinery. The presence of the HCMV DNA at this phase can be shown by applying sensitive techniques such as the polymerase chain reaction (PCR). Contrary to this, during an ongoing active infection when the virions are produced, the resulting viral particles are seen as intranuclear inclusions in the affected cells, also called “the owl eyes”[17]–[20].

A primary HCMV infection leads to a life-long viral persistence. The seroprevalence of HCMV in the general population ranges between 50 to 100% [21]–[24]. The HCMV proteins can be detected in the human tissue in various cellular compartments by means of immunohistochemistry (IHC) [18]. Several reports have demonstrated one or more of these proteins in the neoplastic cells in the GBM and other intracerebral tumours (Table 1). Some of the proteins that have been investigated are components of the tegument or envelope of the virion and are expressed during certain phases of the HCMV life circle (immediate early 1 (IE1, 72 kDa), immediate early 76 kDa protein (clone DDG9), early envelope glycoprotein GP48 (clone QB1/42), early/late protein polymerase processivity factor (p52) (clone CCH2) and the late tegument protein pp65) [1], [8], [11], [25].

**Table 1:** Published reports on expression of human cytomegalovirus in brain tumours.

Kinetic class	Antibody as given in the publication	Antibody number in this study	Tumour type	Grade	Number of cases	% of positive cases	Reference
Immediately	anti-IE1–72	3	Glioblastoma	IV	22	100	[1]
early/Early			Oligoastrocytoma	III	1	100	
			Astrocytoma	II	4	100	
			Meningioma		9	0	
	anti-p52/76kD	5	Glioblastoma	IV	8	100	[1]
			Astrocytoma	II	2	100	
	anti-CMV	4	Glioblastoma	IV	8	0	[9]
	(clones CCH2/DDG9)		Astrocytoma	III	6	0	
			Astrocytoma	II	3	0	
			Oligodendroglioma	II	2	0	
			Ependymoma	II	3	0	
	anti-HCMV (clone E13)		Glioblastoma	IV	81	11	[10]
			Oligodendroglioma	II	20	0	
	anti-p52 (clone CCH2)	4	Glioblastoma	IV	10	0	[10]
	anti-IE	1/2	Glioma		38	0	[12]
			Meningioma		29	0	
	anti-EA (clone QB1/42)	5	Glioma		38	0	[12]
			Meningioma		29	0	
	IE1 antigen (72kDa)	1/2	Glioblastoma	IV	21	100	[8]
			Gliomas	III	12	75	
			Gliomas	I–II	17	82	
	HCMV IEA	1/2	Glioblastoma	IV	10	100	[50]
	HCMV IEA		Glioblastoma	IV	21	95	[25]
	HCMV IE (MAB810)	2	Medulloblastoma	IV	37	92	[7]
	anti-IE1 (MAB810,	2	Glioblastoma	IV	49	16	[44]
	clone 8B1.2)						
	HCMV-IEA (68–72kDa)	1/2	Glioblastoma	IV	80	99	[13]
Late	anti-pp65	8	Glioblastoma	IV	8	100	[1]
			Astrocytoma	II	2	100	
	anti-pp65 (clones 2 and 6)	8	Glioblastoma	IV	8	0	[9]
			Astrocytoma	III	6	0	
			Astrocytoma	II	3	0	
			Oligodendroglioma	II	2	0	
			Ependymoma	II	3	0	
	anti-pp65 (clone 2 and 6)	8	Glioma Meningioma		38 29	0 0	[12]
	HCMV late		Glioblastoma	IV	21	95	[25]
	HCMV late (MAB8127)	6	Medulloblastoma	IV	37	73	[7]
	anti-pp65 (clones 2 and 6)	8	Glioblastoma	IV	49	51	[44]
	HCMV late (47–55kDa)	7	Glioblastoma	IV	80	95	[13]

The proteins produced by the HCMV have been reported to be involved in numerous events, significant for tumour progression. It has been reported that these proteins influence the telomerase activation, angiogenesis, chronic inflammatory environment, immunosuppression, cellular motility and invasion, cell-cycle modulation, and anti-apoptotic effects [21], [26]–[33].

Noteworthy, none or extremely low levels of the HCMV proteins or nucleotides have been reported to be seen in areas of necrosis in tumours, normal brain tissue adjacent to tumour, brain tissue obtained from controls, or subjects suffering from various neurodegenerative diseases [1], [8], [33]. These observations support the notion that the HCMV proteins might be of significance for tumour initiation or progression.

In this study, we have assessed the expression of the HCMV proteins, applying the IHC and tissue microarray (TMA) techniques to a large unselected sample of various intracranial tumours including gliomas, medulloblastomas (MB), primary diffuse large B-cell lymphomas of the central nervous system (CNS DLBCL), and meningiomas. The aim has been to identify the best and most reliable method to assess the expression of the HCMV proteins in surgical samples of tumours and to analyse whether the type of the tumour, grade of the tumour, or the age of the patient influences the obtained expression pattern.

## Materials and Methods

**Figure 1:**
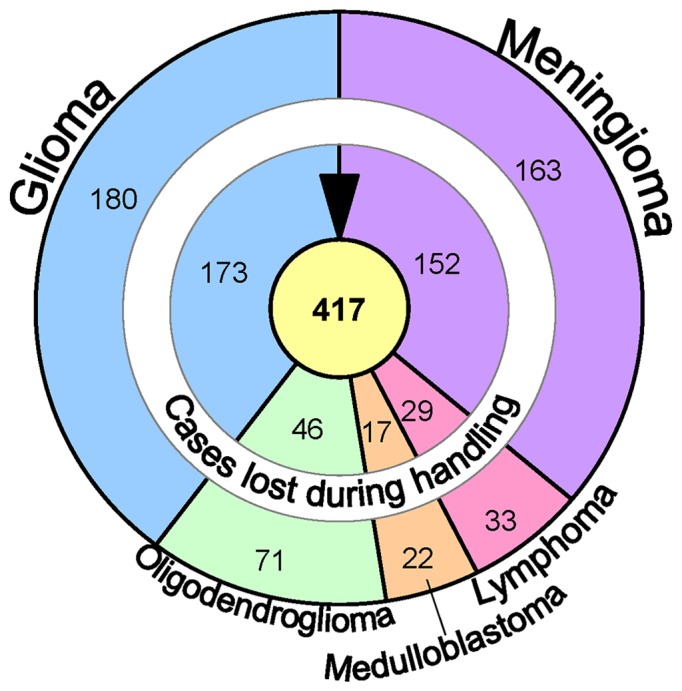
Flowchart summarizing the included cohorts. The outer circle indicates the number of cases at the start, and the centre indicates the number of cases in each cohort available for the assessment of human cytomegalovirus protein expression.

The study was carried out on human brain tissue obtained either during surgery (467 subjects) or postmortem (17 subjects). The original sample included material from subjects with intracranial tumours (469 subjects, Table 2), HCMV infection (one case), and brain tissue from control cases without a neurological disease (n = 14). The subjects included had given their consent for the use of the tissue, and the experimental procedures were approved by the local ethical committees in either Finland or Sweden. Some of the originally included cases were lost during the processing; finally, 417 cases remained for the analysis (Figure 1).

**Table 2:** Demographics of the cases tumor cohorts.

Tumor cohorts	Number of cases	Gender F/M	Age, mean ± SE, (range)	WHO grade/Subtype	Number of cases				
Meningeoma *from Finland*	163	129/34	52±1 (21–86)	I	122				
				II	32				
				III	9				
Lymphoma ^1^ *from Sweden*	33	20/13	62±2 (33–80)	Non-germinal center	25				
				Germinal center	5				
Medulloblastoma *from Sweden*	22	6/16	4±1 (0–16)	Classic	15				
				Nodular	3				
				Anaplastic/large	4				

						Type based on protein expression [38], [55]			
						Classical	Proneural	Mesenchymal	Other
Oligodendroglioma *from Finland^2^*	71	34/37	43±2 (18–76)	II	43	2	36	2	3
				III	15	2	11	1	1
Oligodendrogliomas *from Sweden*	14	6/8	44±4 (27–71)	II	12	1	8	2	1
				III	2	1	-	-	1
Astrocytomas *from Sweden*	133	53/80	52±1 (22–80)	II	43	11	17	5	10
				III	18	5	9	3	1
				IV	72	31	26	14	1
Oligoastrocytom *from Sweden*	33	12/21	40±2 (19–74)	II	21	2	16	2	1
				III	12	2	8	2	-
Low grade gliomas, ∑	128	53/75	41±1 (18–75)						
									
High grade gliomas, ∑	123	52/71	54±1 (22–80)						
									
Gliomas, ∑	251	105/146	47±1 (18–80)						
									
Total	469	260/209	48±1 (0–86)						
									

### Ethical statement

Written consent was obtained from all patients and all material was obtained in a manner compliant with declaration of Helsinki. The use of the tissue for the study was approved by the Uppsala Regional Ethical Review board (Dnrs 2002/330, 2005/542/31/1, 2006/229, 2008/246, 2011/286) and by the Ethical Committee of Kuopio University Hospital (Drns 2003–20, 2003–74). All ethical considerations followed the national legal requirements. The authors report no conflicts of interest.

### Meningioma, low/high grade glioma, and medulloblastoma cohorts

Three cohorts have been described in detail previously: the meningioma cohort from Finland (n = 163 cases) [34], MB cohort from Sweden (n = 22 cases) [35], and the glioma cohort from Sweden (n = 180 cases) [36]. TMA blocks had been constructed for each of these previously published studies, and newly cut sections, 4µm thick, were produced for this study.

In addition, two cohorts not previously described were also included: an oligodendroglioma (ODG) cohort from Finland (n = 71 cases) and a CNS DLBCL cohort from Sweden (n = 33 cases).

### Oligodendroglioma cohort

Patients with an original diagnosis of ODG, anaplastic ODG, or oligoastrocytoma, who underwent an operation between 1982 and 2003, were identified in the register of the Department of Pathology, Kuopio University Hospital. Only primary tumours were selected. Seventy-one subjects were identified. All archived hematoxylin-eosin (HE) stained sections were reassessed, regions to be sampled for the TMA were marked, two 0.6 mm cores were sampled in each case, and TMA blocks were constructed as described previously [37], [38]. Newly cut consecutive sections, 4 µm thick, were placed on SuperFrost Plus slides (Gerhard Menzel GmbH, Braunschweig, Germany) stained for characterization of the tumour and for assessment of the HCMV expression (Table 2).

### Primary diffuse large B-cell lymphoma of the central nervous system (CNS DLBCL)

All CNS DLBCLs within the geographic region served by the Uppsala University Hospital, diagnosed between 1994 and 2011, were retrieved from the archives. A total of 88 subjects were identified. All subjects with a diagnosis of CNS DLBCL following a stereotactic needle biopsy were excluded. Thus, tissue samples from 31 cases obtained during open surgery and two samples obtained at autopsy were included. All original slides were reassessed and two regions for the sampling of core-cylinders with a diameter of 1.0 mm were identified, and the TMA was constructed as previously described [39]. TMA blocks were cut into consecutive 3 µm sections (thinner sections due to shortage of available tissue) and stained for characterization of the tumour and for assessment of the HCMV expression (Table 2).

### Neurologically non-impaired control cohort

The control cohort included 14 neurologically unimpaired subjects. There were nine females and five males; the mean age at death was 61 years (standard error of means 2 years). The brain weight at autopsy ranged from 1,175 to 1,710 grams (mean 1,374±42 g), and the post-mortem delay ranged from 10 to 120 hours (mean 52±7). The neuropathological assessment revealed no vascular alterations or any signs of a neurodegenerative process. The neuroanatomical region selected was the frontal cortex with grey and white matter. The newly cut consecutive 7 µm sections were stained with various HCMV antibodies (Table 3).

**Table 3:** Antibodies listed in alphabetical order, including dilution and pre-treatments applied.

Antibody	Source	Code	Clone	Epitope	PRT	Dilution	Tumorcohort
B-cell lymphoma (BCL)-2 oncoprotein	DakoCytomation	IR614	124	aa41–54	1	RTU	2
BCL-6 oncoprotein	DakoCytomation	IR625	PG-B6p	aa3–484	1	RTU	2
B-lymphocyte antigen CD20	DakoCytomation	IR604	L26	intracytoplasmic	1	RTU	2
BRG1-associated factor 47 (BAF47)	BD Bioscience	612110	25	aa257–359	1	1:250	2
CD3 T-cell co-receptor	DakoCytomation	IR503	Polyclonal	aa156–168	1	RTU	2
CD4 cell-surface glycoprotein	DakoCytomation	IR649	4B12	external domain	1	RTU	2
CD8 cell-surface glycoprotein	DakoCytomation	IR623	C8/144B	C-terminus	1	RTU	2
CD44 cell-surface glycoprotein	Santa Cruz	sc-7297	Polyclonal	Full-length	2	1:100	1
CD44 cell-surface glycoprotein	DakoCytomation	M7082	DF1485	Full-length	1	1:100	2
CD79α phosphoprotein	DakoCytomation	M7050	JCB117	extracellular	1	1:500	2
Cytomegalovirus (CMV) #1	Millipore	MAB8131	6F8.2	IE of 68–72kDa	3	1:2000	3, 4
CMV #2	Millipore	MAB810R	8B1.2	IE of 68–72kDa	3	1:1000	3–5
CMV #3	BioGenex	MU254-UCE	BM204	EA of 68kDa	3	1:25	3, 4
CMV #4	DakoCytomation	M0854	CCH2+DDG9	IE/EA	3	1:50	3, 4
CMV #5	Novocastra	NCL-CMV-EA	QB1/42	EA	3	1:25	3, 4
CMV #6	Millipore	MAB8127	1G5.2	LA	3	1:400	3–5
CMV #7	Chemicon	MAB8126	2D4.2	LA of 47–55kDa	3	1:50	3, 4
CMV #8	Novocastra	NCL-CMVpp65	2 and 6	C-terminus of pp65	3	1:200	1–8
CMV #9	Novocastra	NCL-CMV-LA	QB1/06	LA	3	1:50	3, 4
Epidermal growth factor receptor	Invitrogen	28–0005	31G7	extracellular	4	1:100	1,2
Epithelial membrane antigen	DacoCytomation	M0613	E29	Full-length	2	1:100	6
Glial fibrillary acidic protein	DakoCytomation	Z0334	Polyclonal	Full-length	5	1:500	1
Human leukocyte antigen –DR	DakoCytomation	M0746	TAL.1B5	33kDa α-chain	6	1:30	2
Isocitrate dehydrogenase 1	Dianova	DIA H09	H09	aa125–137	3	1:500	1,2
Ki67	DakoCytomation	M7240	MIB-1	345 and 395 kDa	2	1:100	1,2
Latent membrane protein of Epstein-Barr virus	DakoCytomation	M0897	CS.1–4	C-terminus	1	1:50	2
Microtubule-Associated protein 2	Sigma-Aldrich	M4403	HM-2	Full-length	2	1:500	1,2
Multiple myeloma oncogene 1	DakoCytomation	IR644	MUM1p	Full-length	1	RTU	2
Neprilysin, CD10	DakoCytomation	IR648	56C6	external domain	1	RTU	2
Oligodendrocyte transcription factor 2	Abnova	H00010215-M03	3C9	Full-length	2	1:100	1,2
Platelet derived growth factor receptor α	Santa Cruz	sc-338	Polyclonal	C-terminus	3	1:100	1
proto-oncogene tyrosine-protein kinase MER	Novus Biologicals	NB 110–57199	Y323	N-terminus	2	1:50	1
Synaptophysin	DacoCytomation	M7315	SYNAP	C-terminus	2	1:50	8
Tumour protein 53	DakoCytomation	M7001	DO-7	Full-length	2	1:50	1,2

IE- immediately early antigen; EA – early antigen; LA-late antigen; PRT- Pretreatment: 1– DAKO systems FLEX TRS high; 2– heat pretreatment, Citrate Buffer, pH 6.0; 3– heat pretreatment, Tris-EDTA, pH 9.0; 4– proteinase K; 5–0.03% protease XXIV; 6– DAKO systems FLEX low. RTU – ready to use; Assessment cohorts 1– oligodendrogliomas, 2– primary central nervous system lymphoma, 3– CVM infected case, 4– test tissue microarray, 5– controls, 6– meningiomas, 7– gliomas, 8– medulloblastomas.

### Human cytomegalovirus antibodies

Nine commercial antibodies (Ab) from 5 different suppliers were selected and tested. According to the manufacturer, these Abs were reported to recognize immediately early, early, or late HCMV antigens (Table 3). Each Ab was systematically tested to determine the optimal staining conditions, i.e., dilution and antigen retrieval method (ARM) required for best results. The ARM applied included heat pretreatment strategies: citrate buffer (10 mM sodium citrate, 0.05% Tween 20, pH 6.0) or Tris-EDTA (10mM tris-base, 1 mM EDTA, 0.05% Tween 20, pH 9.0) and two different enzymatic strategies: proteinase K (ready to use, Dako Cytomation, Glostrup, Denmark); and 0.03% proteinase XXIV (Sigma-Aldrich, St. Louis, MO).

The test stainings were performed on the verified HCMV infected brain tissue (performance of the Abs), on a test-TMA, including tissue cores measuring 2-mm in diameter from different brain tumours, i.e., GBM, MB, anaplastic astrocytoma, anaplastic ODG, ependymoma, and ganglioglioma (performance of Abs and pretreatment requirements) as well as on control brain tissue (staining of normal non-neoplastic cells).

All stained sections were assessed using a light microscope at magnifications x100 to x400. Immunoreactivity (IR) in a defined cellular compartment (nuclear and cytoplasmic) was denoted, and the intensity of the signal was assessed as follows: 0– no IR observed; 1– weak IR, must be investigated at a higher magnification (x400); 2– IR is obvious at high magnification (x200); and 3– IR is obvious at low magnification (x100). The Ab to be chosen was expected to fulfil the following criteria: 1– robust IR and 2– sparse background staining to allow for secure recognition of the specific IR.

### Immunohistochemistry

All stains (except for the CNS DLBCL-classification) were carried out manually. Briefly, unspecific binding sites were blocked with Background Sniper (Biocare Medical, Concord, CA) for 10 min, and sections were incubated with primary Ab overnight at 4°C. PowerVision detection system (Immunologic, Duiven, The Netherlands) was used with a Romulin AEC or DAB chromogen kit (Biocare Medical) for antigen detection. The newly cut sections of the included TMAs were stained with the selected HCMV Abs (Table 3). The newly cut sections of the CNS DLBCL block were stained using the Dako Autostainer Plus (DakoCytomation) with Abs selected for diagnostics (Table 3). Dako EnVision FLEX detection system (DakoCytomation) was used subsequently for the visualization of the staining results, according to the manufacturer's instructions.

### Light microscopic viewing

A case was included when at least 50% of the total area of at least one core sample remained on the slide and if the core was representative of the tumour. The sections were assessed at magnifications x40 to x400 several times by at least two evaluators blinded to the original diagnosis; thereafter, a consensus score was ascribed to each TMA core. The HCMV-IR was assessed and the results were dichotomized as being seen or not seen.

Photographs were taken with an Olympus BX46 microscope, equipped with an Olympus DP72 digital camera, and images were acquired using “cellSens Entry” acquisition software (Olympus Optical, Tokyo, Japan). IBM SPSS Statistics 20 (IBM Corporation, Armonk, NY) was used for statistical analysis.

## Results

### Oligodendroglioma cohort

The TMA included 71 subjects with ODG (Table 2). The male/female ratio was 1.0 and the age at operation ranged from 18 to 76 years (mean ± standard error of means 43±2 years). The majority of these tumours were of the WHO grade II (73%). Tumours were subtyped into protein defined subtypes as described previously, applying the IHC technique [36]. The majority of the subjects represented the protein expression profile reminiscent of the proneural type, 81% [40]. The classical and mesenchymal subtypes represented 7 and 5 percent, respectively. In four cases (7%), no high expression of any of the proteins analysed was observed. Thirteen cases were excluded due to a lack of representative tissue in the TMA.

### Primary diffuse large B-cell lymphoma of central nervous system cohort

The TMA assessed here included thirty-three subjects with CNS DLBCL (Table 2). The male/female ratio was 0.7 and the age at operation ranged from 33 to 80 years (mean ± standard error of means, 62±2 years). Three cases were excluded due to a lack of tumour tissue in the TMA core. The subclassification of the CNS DLBCL followed the WHO classification of Tumours of Haematopoietic and Lymphoid Tissues, 4:th Edition from 2008 [41]. Briefly, germinal center-like (GC) type CNS DLBCL fulfilled the criteria of either expressing CD10 in>30% of the tumour cells or being BCL6 positive and CD10/MUM1 negative. All other cases are considered as being of non-germinal center-like (non-GC) type [42], [43]. According to this classification, 83% of the CNS DLBCL were non-GC and 17% were the GC type.

### Validation of the human cytomegalovirus antibodies

**Figure 2:**
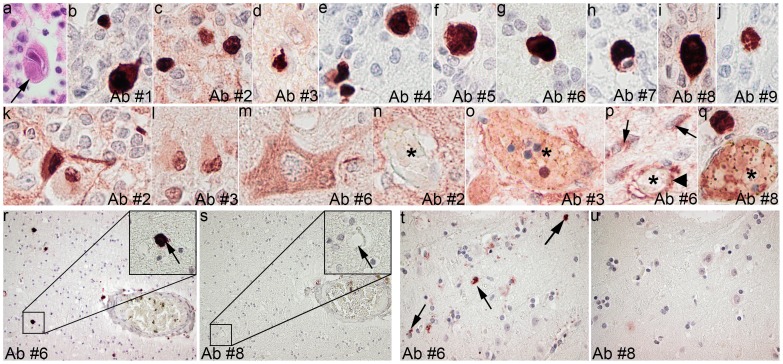
Lesions seen in the brain tissue in a case with a verified active cytomegalovirus (HCMV) infection and in normal brains applying commercial antibodies. (a) The large cell with the “owl eye” inclusion (arrow) seen in the hematoxylin and eosin stain in a case with an active HCMV infection. (b-q) Immunoreactive (IR) and (r-u) IR lesions in normal brain applying the nine commercial antibodies (Ab). Note the labelling of nuclear inclusions in b-j and q; of the nuclei (k) or cytoplasm (m) of neurons; nuclei and cytoplasm of astrocytes (l); neuropil (n) around a vessel (*) with unstained erythrocytes; plasma and some blood cells (o,q) within a blood vessel (*); pericytes (arrowhead) surrounding unstained erythrocytes and cytoplasm of glia cells (arrows) (p). Labelling of the corpora amylacea (r) with antibody #6 and lack of a similar labelling (s) with Ab #8 (arrows). Labelling of the glial cytoplasm (arrows) (t) with Ab #6 and lack of a similar labelling (u) with Ab #8. Magnification x400 (a-q, t, u) and x200 (r, s) Abs used immediately early antigen: #1 –clone 6F8.2, #2– clone 8B1.2, #3– clone BM204; #4– immediately early + early antigens, clones CCH2+DDG9; #5– early antigen clone QB1/42; late antigen: #6 –clone 1G5.2, #7 –clone 2D4.2, #8– pp65, clones 2 and 6, and #9– clone QB1/06.

The HCMV positive case was a male foetus, gestational age 30 weeks, and the autopsy was carried out 24 hours postmortem. The HCMV infection was verified by PCR carried out on the foetal blood and a postmortem DNA analysis, confirming the HCMV infection, which was carried out on the samples taken from the lung and liver tissue. This case was assessed using all the selected nine commercial HCMV Abs (Table 3). The neuropathological assessment of the HE stained sections revealed multifocal inflammation and numerous cells with inclusions. IR of these inclusions was observed with all the tested HCMV Abs (Figure 2). Noteworthy, different staining patterns were observed upon the application of different Abs (Figure 2). In addition to labelling of the inclusions, some Abs labelled the nucleus or the cytoplasm of the neurons (Ab #2, 4, 5, 6, and 8), nucleus or cytoplasm of the glia cells (Ab #3, 6), and neuropil (Ab #1, 2). Labelling of the red blood cells and pericytes (Ab #3, 5, 6, and 8) was also noted. No distinct pattern could be seen related to the protein kinetics regarding the compartment of labelling, i.e., blood, pericyte, cytoplasm, and nucleus.

### Optimization of staining

**Figure 3:**
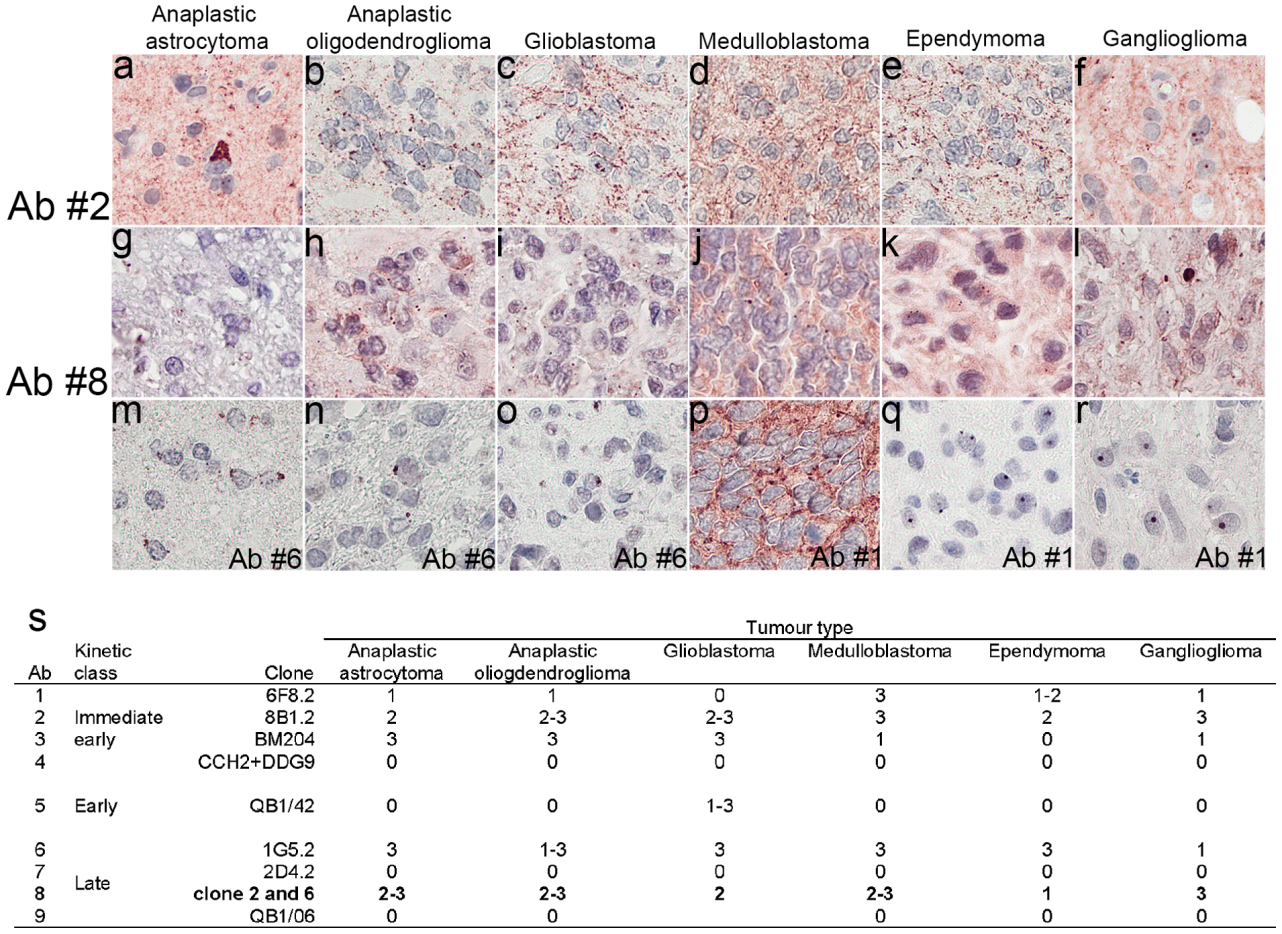
Human cytomegalovirus (HCMV) immunoreactive (IR) grains in tumour cells, seen upon the application of different commercial antibodies (Ab). Note the prominent background labelling with Ab #2 in the astrocytomas when compared with results applying Ab #6 and #8. Magnification x400. The results assessing the extent of the labelling while applying the nine different antibodies are shown in s. 0– no IR; 1– weak IR, must be viewed at a high magnification (x400); 2– IR is obvious at high magnification (x200); and 3– IR is obvious at a low magnification (x100).

Results applying all nine commercial HCMV Abs on the test TMA with cores of various brain tumours are shown in figure 3. No IR was observed while applying three of the listed Abs (Ab #, 7, and 9) independent of the ARM or dilution used. With the remaining six Abs, the IR was seen as granules of different diameters localized to the nucleus and/or the cytoplasm of the tumour cells (Figure 3). The number and diameter of these granules as well as the background IR varied significantly when comparing different Abs. The use of three Abs (Ab #2, #6 and #8) resulted in an intense labelling of the granules in the majority of the tumours analysed. While applying Ab #2, significant background IR was observed both in the cytoplasm and in the neuropil, interfering with the assessment of the “specific” labelling. In contrast, background labelling while applying Ab #6 and #8 was relatively low and did not interfere with the assessment of the IR granules, which were easily recognizable.

### Staining of non-neoplastic brain tissue

Control staining was carried out on the normal brain tissue and included three Abs (#2, #6 and #8) that performed best on the test TMA, including various tumours. Ab #6 was noted to label corpora amylacea, cytoplasm of the pericytes, and cytoplasm of the glial cell in the control tissue (Figure 2) whereas Abs #2 and #8 did not label any of the cellular structures.

Thus, based on the results obtained while assessing the infected brain tissue, normal brain tissue, and tumours (Figures 2 and 3), Ab #8, directed to late protein pp65, was considered to perform best; thus, this Ab was chosen to be applied on the tumour cohorts.

### Tumour cohorts and HCMV

Expression of the HCMV protein pp65 was analysed in 417 intracranial tumours in the current study, wherein 198 cases were from Eastern Finland and 219 cases were from central Sweden (Table 4). The male/female ratio was 0.8, and the age ranged from 0 to 86 years (mean ± standard error of means 48±1). There were 152 extra-axial and 265 intra-axial tumours. The cohort included five different subgroups. There were 173 astrocytomas (male/female ratio 1.6; mean age 49±1), 46 ODGs (male/female ratio 1.3; mean age 42±2), 17 MBs (male/female ratio 3.3; mean age 3±1), 29 CNS DLBCLs (male/female ratio 0.6; mean age 62±2), and 152 meningiomas (male/female ratio 0.3; mean age ± standard error of means 52±1 years). The routine HE stain did not reveal classical HCMV inclusions in any of the tumours investigated here. The results obtained while applying the IHC technique are summarized in Table 4.

**Table 4:** Expression of cytomegalovirus protein pp65 in the included extra and intra axial brain tumors.

Tumour cohorts	WHO grade/Subtype	Number of cases	% of HCMV positive cases				
Meningeoma *from Finland(Fin)*	I	113	87				
	II	31	90				
	III	8	75				
	Meningeomas Fin	152^+11^	87				
Lymphoma *from Sweden (Sw)*	Non-germinal center	24	79				
	Germienal center	5	60				
	Lymphomas Sw	29^+1^	76				
Medulloblastoma *from Sw*	Classic	10	70				
	Nodular	3	33				
	Anaplastic/large cell	4	75				
	Medulloblastomas Sw	17^+5^	65				

				Type based on protein expression [38], [55]			
				Classical	Proneural	Mesenchymal	Other
Oligodendroglioma *from Fin*	II	36	100	100_1_	100_32_	100_2_	100_1_
	III	10	90	100_2_	86_7_	-	100_1_
	Oligodendrogliomas Fin	46^+12^	98	100	97	100	100
Oligodendroglioma *from Sw*	II	12	92	0_1_	100_8_	100_2_	100_1_
	III	2	50	0_1_	-	-	100_1_
	Oligodendrogliomas Sw	14	86	0_2_	100	100	100
Astrocytoma *from Sw*	II	41	80	82_11_	87_15_	40_5_	90_10_
	III	18	89	80_5_	89_9_	100_3_	100_1_
	IV	68	90	84_31_	96_23_	92_13_	100_1_
	Astrocytomas Sw	127^+6^	87	83	91	81	92
Oligoastrocytom *from Sw*	II	21	95	50_2_	100_16_	100_2_	100_1_
	III	11	100	100_2_	100_7_	100_2_	-
	Oligoastrocytomas Sw	32^+1^	94	75	100	100	100

HCMV- human cytomegalovirus, superscript- number of not assessable cases, subscript – number of assessed cases in the group.

Eighty-seven percent of the astrocytomas were HCMV-IR and 90% of GBMs. The IR was independent of the protein defined subtype. Out of the five available WHO grade II astrocytomas of mesenchymal subtype, 60% were HCMV negative. Sixty-five percent of the MBs were HCMV-IR (classical 70%, anaplastic/large cell 75%, and nodular 33%). The ODG cohort from Finland showed HCMV-IR in 98% (100% grade II and 90% grade III) and the ODG cohort from Sweden showed 86% IR (92% grade II and 50% grade III). Most subtypes defined by the protein expression were HCMV-IR. There were two cases out of five available classical ODGs grade II/III (40%) that were HCMV negative.

In conclusion, 86% of all low-grade (WHO II) gliomas from Sweden (n = 74) and 100% from Finland (n = 36) were HCMV-IR. Ninety percent of the high-grade (WHO III, IV) gliomas from Sweden (n = 99) and 90% from Finland (n = 10) were IR. Thus, out of the 219 gliomas analysed here, 90% were HCMV-IR.

Seventy-six percent of the CNS DLBCLs were HCMV-IR, and the labelling was higher in non-GC like (79%) when compared to GC-like (60%) subtype. Eighty-seven percent of the meningiomas were HCMV-IR, and the IR was independent of the WHO grade (87% IR in grade I, 90% IR in grade II, and 75% IR in grade III).

**Figure 4:**
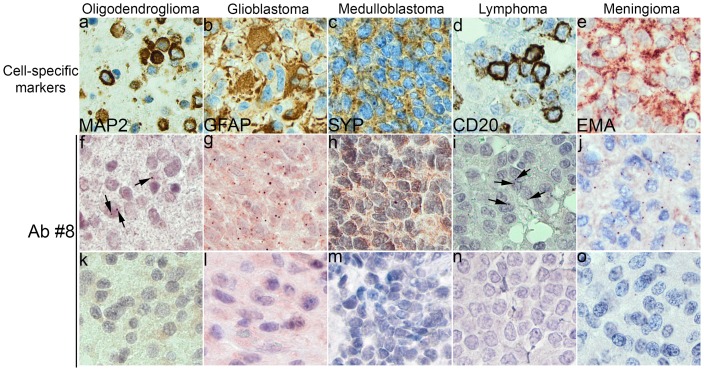
Immunoreactive (IR) grains in the tumour cells, seen upon the application of the late human cytomegalovirus tegument protein antibody pp65 (clones 2 and 6) in f-j. Identification of cell type was carried out applying cell type specific antibodies (Ab) – a) membranous labelling of oligodendrocytes with Ab directed to microtubule associated protein 2 (MAP2); b) cytoplasmic labelling of astrocytes with Ab directed to glial fibrillary acidic protein (GFAP); c) granular cytoplasmic labelling of neuronal cells with Ab directed to synaptophysin (SYP); d) membranous labelling of lymphocytes with Ab directed to B-lymphocyte antigen CD20 (CD20) and e) cytoplasmic labelling of meningothelial cells with Ab directed to epithelial membrane antigen (EMA). HCMV-IR grains seen in all tumour types (f-j), few pp65-IR granules in the ologodendroglioma and lymphoma samples (arrows on f and i) and numerous granules in glioblastoma, medulloblastoma and meningioma (g, h, j). In k-o cases lacking HCMV-IR. Magnification x400.

The observed HCMV-IR in the tumours assessed here was seen as granular staining in the nucleus or in the nucleus and cytoplasm of the neoplastic cells. The size and the quantity of the IR granules varied significantly between the subjects and between the tumour types (Figure 4). In ODGs, small dot-like granules were often observed whereas the granules observed in the GBM or MBs were both larger and more irregular in shape. Noteworthy, GBM, MB, and meningioma displayed the highest number of grains in a core sample whereas the CNS DLBCL displayed only a few HCMV-IR cells scattered in the sample. Noteworthy, there was a substantial variation in the regional distribution of the positive granules in a core. Furthermore, the number of HCMV-IR cells varied from one IR cell/core to virtually all tumour cells in a core being IR (Figure 4).

## Discussion

In this study, we have assessed the expression of the HCMV protein in a large cohort of intra- and extra-axial brain tumours. The cohort in our study included in total 417 tumours of various WHO grades, representing gliomas of various types, MBs, CNS DLBCL, and meningiomas. We noted that the HCMV protein pp65 expression was indeed common in brain tumours and ranged from 65 to 98 percent. This expression seemed to be independent of the tumour type, grade, patient age, and gender.

It is well known that the choice of Ab and sensitive staining protocols are of significance while assessing the HCMV proteins. The previously published results have reported that the HCMV proteins have been seen in 0 to 100% of the assessed tumours [1], [7]–[10], [12], [13], [25], [44]. We tested nine different commercial Abs directed toward different HCMV proteins, including Abs that detect proteins that are expressed at the early stage of the viral cycle to those that are expressed at the later stages. It should be noted that it is difficult to compare our results in detail with those previously published, as previous publications do not always provide details (clone) regarding the used antibodies. In most papers, only the antigen (immediately early/early/late) and the manufacturer producing the Ab are stated. In our study, all of the Abs tested that are commercially available were efficient when assessing the HCMV infected tissue. The hallmark lesions of an HCMV infection, including the inclusions seen in the HE stain, were repeatedly labelled. Thus, all of the Abs applied here can readily be used in the assessment of tissue with an active HCMV infection. In addition, however, labelling of glial cells, neurons, pericytes, erythrocytes, and neuropil was seen to various extents. This labelling seen is difficult to interpret on its own, without inclusions. Thus, the IR as such is not sufficient to verify an active infection. It is noteworthy that some of the tested Abs when applied on the control, uninfected brain tissue, labelled the glial cells, corpora amylacea, pericytes, and red blood cells. Whether these findings are a sign of a latent HCMV infection cannot be ruled out. In general, the choice of the Ab to be used while assessing IR is of great importance. It has previously been shown that applying various Abs to assess the same protein might lead to various results that are interpreted by the assessor in different manners, leading to controversies [45]. Here, to be on the safe side, we excluded all Abs that displayed a strong background staining or stained various cells and cell compartments (nucleus vs. cytoplasm) in the normal brain tissue. This scrutinized approach meant that only one Ab out of the nine tested fulfilled our requirements and was thus chosen. Consequently, this strict approach made us confident that what was detected as IR was indeed the HCMV late protein pp65 in the cells. The specificity of this Ab is also supported by previous report indicating that the Ab #8 recognizes a protein of the expected molecular mass [46].

Our results applying the Ab #8 regarding GBMs and MBs are in line with previously published reports applying the Abs #3, #4, and #6 [1], [7], [9]. In contrast, our results regarding GBM differ in comparison with some of the previously published reports while applying Abs #5, #7, and #8 [9], [12], [13]. This difference, particularly regarding Ab #8, is probably due to the staining protocol applied as has already been discussed in a previous publication [8]. Furthermore, dilution, ARM, and the use of a detection system, parameters that are well known to influence the IHC outcome, are of significance [47], [48]. Here, we carried out a systematic testing applying various dilutions and ARMs to obtain optimal results. Our testing led to the use of heat pretreatment, i.e., pressure cooker with a high pH buffer, as ARM. Others have implemented heat pretreatment with a low pH buffer or enzymatic digestion, ARMs not optimal in our study. Furthermore, the detection system used here, which was polymer based, has high signal amplification (PowerVision, Immunologic) [49]. Others have used different detection systems, including the polymer based [44] or the three-step labelled streptavidin-biotin method [1], [7], [13], [50]. All of these methods are in principle designed to amplify the labelling signal. Thus, due to the different approaches, comparing our results with those previously obtained is not fully possible. Even the thickness of the section has been discussed as being of importance if the staining method used is not sensitive enough [8]. Here, we used the thickness of sections applied in routine diagnostics ranging from 3 to 7 µm, without noting any major differences in the obtained results. The advantage of using thin sections is being able to assess the individual cells rather than the cell masses. A number of additional factors such as fixative, fixation time, and type of embedding medium have been demonstrated in several earlier studies to be of importance for the IHC outcome [51]–[53]. All tumour samples assessed here were, in principle, processed using the fixative and fixation time in a similar way. Although the paraffin used and the dehydration system in Finland and Sweden differed, we did not note any significant influence of these factors on the IHC results.

Thus, based on our observations, the Ab directed toward the HCMV late protein pp65 could readily be used on the formalin fixed tissue, independent of the thickness of the section or the routine handling of the sample (fixative, fixation time, paraffin), whereas one should be cautious regarding the ARM as well as the detection system.

Here, we only assessed the protein expression applying the IHC method. Some previous studies have also carried out the HCMV DNA and/or RNA analysis on the tissue investigated in addition to the IHC analyses [1], [7], [8], [12], [13], [44]. It is worth noting that while applying the DNA and/or RNA –assessment, fewer cases are positive when compared with the IHC-detection of the HCMV protein expression [11], [54]. This quite surprising result might be related to the observation that the HCMV-IR was patchy, that is, unevenly distributed in the tissue section. Thus, while applying different methods in assessing the HCMV, the sampling strategy might significantly influence the obtained results.

Presence of the different HCMV proteins in the gliomas has been analysed earlier in a number of studies [1], [8]–[10], [12], [13], [25], [44], [50]. Several publications, in line with our results, have indicated that the HCMV proteins are indeed frequently present in the GBM [1], [8], [13], [25], [44], [50]. The IR for late protein pp65 in our study was similar to previous reports; however, in addition to the GBMs the pp65 protein was also detected in the low-grade gliomas. Overall, 90% of all the gliomas independent of the WHO grade were HCVM-IR. Furthermore, although not previously reported but based on our results, the molecular subtype of the tumour did not influence the HCMV protein expression.

Medulloblastomas, preferentially seen in children, showed HCMV IR in 65% of the cases. This is in accordance with a previous report describing that 92% of the medulloblastomas expressed early and 73% expressed late proteins [7].

To our knowledge, the analysis of the HCMV in the CNS DLBCL has never been performed previously. In the current study, CNS DLBCL, a high malignant brain tumour, was IR in 76% of the cases. As MBs, these tumours are highly proliferative and subjects with this tumour have an extremely poor prognosis and a short survival time.

Interestingly, contrary to a previous report [1], we noted that 87% of the WHO grade I meningiomas (tumours considered benign with good prognosis) showed the HCMV-IR. In the WHO grade II meningiomas, the percentage was even higher, 90%. The lower HCMV-IR in the WHO grade III meningiomas (75%) could be due to a low number of cases included (n = 8). Our differing results when compared to the previous reports are probably due to selection bias and methodology. In one of the reports, all types of brain tumours were negative, disputing the methods used, and the other study included only nine cases [1], [12]. Thus, our results are in principle unique, indicating that the HCMV protein is expressed in these tumours of low malignancy. It is, however, well known that a meningioma has a high tendency to recur if not extirpated in total, indicating a good “survival” tendency of the tumour cells.

Here, we did not assess systematically the extent of the labelling in each case as has been carried out by other investigators [13]. The labelling, however, was seen in a sample measuring 0.6 to 2 mm in diameter (TMA core), indicating that a dichotomized assessment of the protein does not seem to be influenced by the sampling deficit. However, the distribution of the HCMV-IR might be, as we noted, patchy and uneven. This should be considered while assessing the extent of the HCMV-IR in routine diagnostics. It is common to obtain surgical samples ranging from a few mm (needle biopsy) to several cm (surgical resections); thus, the sampling deficit might be of significance.

We have been unable to assess any potential prognostic aspects related to the expression of the HCMV protein since the operation techniques varied (partial/total resection) and different chemo- and radiotherapy regimens had been applied. However, it is noteworthy that tumours ranging from the WHO grade I to WHO grade IV of various cellular origins and with extremely different proliferation rates (1 up to 90 percent) have all expressed the HCMV protein related to the late stage of the viral cycle.

Signs of an ongoing HCMV infection such as inclusions in the HE or IHC stain were never seen in any of our cases. Thus, we lack proof for an activation of a latent viral infection in this tumour population. What was detected was the expression of the late HCMV protein pp65 known as the tegument protein. This protein can be produced in excess in cells during an active symptomatic/asymptomatic infection and assembled in particles called dense bodies that lack viral DNA. The HCMV seropositivity is high in adults, and it is presumed that most of us have suffered from this infection even unknowingly, asymptomatic. Based on previous studies, the viral DNA can be detected in monocytes whereas the late viral proteins such as pp65 are common in tissue samples from patients with the HCMV infection. Whether all our subjects with the expression of late HCMV protein pp65 in the tumour cells have monocytes harboring the latent HCMV virus is not assessed here. This possibility however cannot be neglected due to the high levels of the HCMV seroprevalence, especially in adults [23], [24], [55]. It is also known that the ability of the HCMV to replicate in the monocytes, which harbor the latent HCMV, is dependent on the state of the cellular differentiation [56], [57]. Interesting to note, however, is that most studies assessing the activation of latent HCMV are carried out *in vitro,* and *in vivo* tumour conditions are in general difficult to replicate. Meanwhile, only a few cell types propagate the HCMV *in vivo* and the GBM cell lines are among them [18].

Some studies have reported that the HCMV proteins are present not only in the brain tumours but also in other types of cancers (skin, breast, colorectal, prostate) [2]–[4], [58]. Based on our results indicating that the late HCMV protein pp65 is present in a wide range of different tumour types within the skull, it emphasizes that further studies assessing the HCMV protein in various pathological conditions are warranted.

Recently, a number of studies have indicated that the anti-HCMV drug treatment can alter the outcome of the GBM in human, animal models, and cell cultures [15], [16], [59]. These drugs, at least two of them, induce apoptosis and thus influence the survival of the tumour cells. Interestingly, one of the drugs is dependent on the viral DNAase (assessed on human material) [15], [59] whereas the other is not (assessed on mouse models and cell cultures) [16]. Surprisingly, both the HCMV-expressing and non-HCMV-expressing tumours, when assessed in an experimental design, seemed to be influenced while using the drug, independent of the the viral DNAase [16].

In conclusion, we systematically analysed the performance of nine commercial HCMV-Abs on the brain tissue samples obtained from a verified HCMV infected patient, from 14 neurologically unimpaired subjects lacking pathology, and on a set of various brain tumours in TMA. The best performing Ab, the late HCMV protein pp65 (clones 2 and 6) was further used to assess the HCMV expression in different extra- and intra-axial brain tumours. This late HCMV protein pp65 was detected in all types of tumours analysed, and the IHC expression did not depend on the patient's age, gender, tumour type, or grade. The labelling pattern observed in the tumours differed from the labelling pattern observed in the tissue with a verified active HCMV infection. No signs of an active HCMV infection were noted; thus, we do not feel confident in using the term “HCMV infection” but rather implement the term “expression of late HCMV protein pp65” in tumour cells, which certainly might be a sign of a latent HCMV infection.
